# Light Trapping Effect in Perovskite Solar Cells by the Addition of Ag Nanoparticles, Using Textured Substrates

**DOI:** 10.3390/nano8100815

**Published:** 2018-10-10

**Authors:** Jiabin Hao, Huiying Hao, Jianfeng Li, Lei Shi, Tingting Zhong, Chen Zhang, Jingjing Dong, Jie Xing, Hao Liu, Zili Zhang

**Affiliations:** 1 School of Science, China University of Geosciences, Beijing 100083, China; jiajooy@163.com (J.H.); m18810623195@163.com (J.L.); 2019170012@cugb.edu.cn (L.S.); zhongtingtingabc@163.com (T.Z.); zhangchenphysics@163.com (C.Z.); jjdong@cugb.edu.cn (J.D.); xingjie@cugb.edu.cn (J.X.); 2014010007@cugb.edu.cn (H.L.); 1987010158@cugb.edu.cn (Z.Z.); 2 School of Materials Science and Technology, China University of Geosciences, Beijing 100083, China

**Keywords:** perovskite, textured-FTO, Ag NPs, light trapping, LSPR

## Abstract

In this contribution, the efficiencies of perovskite solar cells have been further enhanced, based on optical optimization studies. The photovoltaic devices with textured perovskite film can be obtained and a power conversion efficiency (PCE) of the textured fluorine-doped tin oxide (FTO)/Ag nanoparticles (NPs) embedded in c-TiO_2_/m-TiO_2_/CH_3_NH_3_PbI_3_/Spiro-OMeTAD/Au showed 33.7% enhancement, and a maximum of up to 14.01% was achieved. The efficiency enhancement can be attributed to the light trapping effect caused by the textured FTO and the incorporated Ag NPs, which can enhance scattering to extend the optical pathway in the photoactive layer of the solar cell. Interestingly, aside from enhanced light absorption, the charge transport characteristics of the devices can be improved by optimizing Ag NPs loading levels, which is due to the localized surface plasmon resonance (LSPR) from the incorporated Ag NPs. This light trapping strategy helps to provide an appropriated management for optical optimization of perovskite solar cells.

## 1. Introduction

Organic–inorganic halide perovskite semiconductors, as the most potential absorber materials for the photovoltaic applications, have attracted growing attention due to their high light absorption coefficient [1], tunable band structure [2,3], high charge carrier mobility [4,5], and low fabrication cost. A rapid improvement in perovskite solar cells has been witnessed based on the substantial and further understanding in materials and device. Recently, much research on the enhancement in performance and efficiency of perovskite solar cells have been brought through the optimum deposition technique of perovskite layer as well as the interface quality enhancement [6,7,8,9,10]. However, keeping a balance between generating carriers and extracting carriers in perovskite solar cells is still a challenge. On the one hand, a thick absorption layer should be used to meet the requirement of sufficient adequate light absorption, which is necessary for generating more photo carriers. On the other hand, the thickness of the absorption layer should generally be less than the diffusion length of carriers to efficiently collect carriers, which means that a thinner active layer is favorable. Light trapping design has been proved to be an effective way to solve the balance problem in silicon thin film, organic and dye-sensitized solar cells [11,12,13]. However, there is a lack of intensive studies of light trapping effects in perovskite solar cells.

One of the light trapping ideas is that the random reflection/diffraction of light by the textured topography leads to internal reflection in photovoltaic devices, which has been realized in silicon thin film solar cells by using textured substrates [14]. Numerical simulation results showed that the maximum gain for this kind of “light trapping” in a textured film is 4*n*^2^, where *n* is the index of refraction of the film [15]. Based on a large number of experimental results, such a light trapping technique has greatly contributed to the improvement of performance of silicon thin film solar cell. [16,17]. As for perovskite solar cells, there are few studies on this kind of light trapping technique. Most recently, Marcos Soldera proposed an optical model to calculate light absorption in perovskite layer on sine-shaped patterned indium tin oxide (ITO) substrates. The result showed that a 200 nm thick perovskite layer on the textured ITO is sufficient to achieve nearly the same light absorption as in a flat, 500nm thick perovskite absorber [18]. However, there is still a lack of experimental evidence.

The other light trapping idea is surface plasmon resonance (SPR) induced by metal nanoparticle [11,19,20]. Plasmonic enhancement in solar cells is mainly attributed to (i) radiative effects in which light scattering and electromagnetic near-field enhancement to increasing effective absorption, and (ii) non-radiative effects where hot-electron transfer and plasmon resonant energy transfer contributes to improved photocurrent generation [21,22]. The plasmonic light trapping idea has been wildly used in silicon, organic and dye-sensitized solar cells. Recently, several attempts have been made on using SPR in perovskite solar cell [23,24,25,26]. Lu et al. introduced the irregular Au-Ag alloy “popcorn-shaped” nanoparticles into mesoporous TiO_2_ layer, and optical absorbance of perovskite film in the range of 580 nm to near infrared was enhanced, which was mainly due to the light trapping effect by the SPR of popcorn NPs [23]. Mali et al. used an electrospinning technique to fabricate TiO_2_ nanofibers with embedded Au NPs to increase photocurrent in perovskite solar cells, and they attributed the enhancement to the SPR effects of Au NPs [24]. Huang et al. used rationally-designed Au@Ag core-shell nanocuboids as an improved light harvesting strategy to improve photo-to-electron conversion efficiency over the entire visible range [25]. It is proved that the localized surface Plasmon resonance effect and the strong scattering effect of Ag@SiO_2_ can enhance the efficiencies of perovskite solar cells [26]. However, intensive and systemic study is still needed.

Herein, we introduce textured FTO/glass substrates with a larger roughness and a compact TiO_2_ layer that is embedded into a low-cost Ag plasmonic nanoparticles in mesoporous perovskite solar cells simultaneously. As a result, a CH_3_NH_3_PbI_3_ film with textured morphology and large crystal grain size on textured FTO was formed. Moreover, significant light absorption enhancements in devices with incorporating Ag NPs into compact TiO_2_ layers in the long wavelength region (>500 nm), and the surface plasmon resonance from metallic nanostructures can enhance the generation of charge in devices. Therefore, this strategy can not only improve nucleation and growth mechanism of perovskite, but also increase the trap light effect in whole devices, which is crucial to the performance enhancement of perovskite solar cells.

## 2. Materials and Methods

### 2.1. Materials

The transparent conductive FTO/glass substrates (Pilkington) included the smooth FTO/glass substrates (10 Ω/sq, S-FTO) and the textured FTO/glass substrates (20 Ω/sq, T-FTO) were used in this work. The titanium(IV) isopropoxide (99.5% purity) was supplied by Alfa Aesar (Shanghai, China) Chemical Co., Ltd. lead(II) iodide (PbI_2_, 99% purity) was purchased from Sigma-Aldrich Co. (St. Louis, MO, USA) Methylammonium iodide (MAI, ≥99.5% purity), Spiro-OMeTAD (≥99.5% purity), Li-TFSI (≥99% purity) and TBP (≥96% purity) were obtained from Xi’an Polymer Light Technology Crop (Xi’an, China). Silver nanoparticles ethanol solution (DK101-2, 2000ppm) was supplied by Deco Ltd. Co. (Beijing, China). Other materials, including N, N-Dimethylformamide (DMF, ≥99.9% purity) and chlorobenzene (99.5% purity) were purchased from Aladdin Ltd. Co. (Shanghai, China).

### 2.2. Device Fabrication

**Figure 1:**
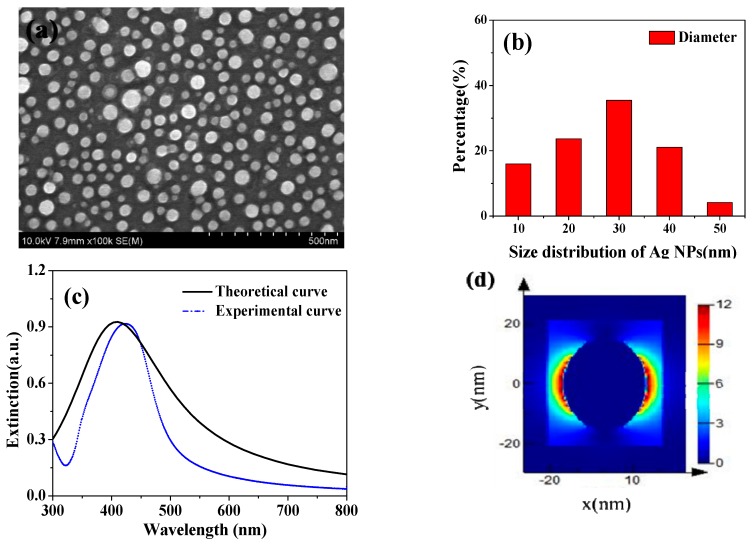
(**a**) SEM image of the Ag nanoparticles in ethanol; (**b**) Size distribution of Ag NPs; (**c**) extinction spectra for the 30 nm diameter Ag NPs; (**d**) Electric field distributions at resonant peak of single Ag NPs.

The structure of the perovskite solar cells is Glass/FTO/compact-TiO_2_(c-TiO_2_)/mesoporous-TiO_2_ (m-TiO_2_)/CH_3_NH_3_PbI_3_/Spiro-OMeTAD/Au, and main fabrication procedure is shown in Figure 1. FTO/glass substrates were cleaned in acetone, isopropanol and ethanol, deionized water sequentially for 15 min and then dried under air stream. A compact TiO_2_ precursor solution consisting of 100 µL titanium (IV) isopropoxide, 2.5 mL ethanol and 20 µL dilute hydrochloric acid stirred for 20 min. The compact TiO_2_ precursor solution was mixed with an Ag nanoparticles ethanol solution to fabricate plasmonic sensitized photoanode. The Ag nanoparticles with 0, 5%, 6.7%, 10%, 20% and 33.3% in mass proportion were stirred for 25 min, which was spin-coated on the smooth transparent and the textured conductive Glass/FTO substrates at 3000 rpm for 30s and subsequently sintering at 500 °C for 30 min. The substrates were further treated with 40 mM TiCl_4_ aqueous solution at 70 °C for 30 min in the oven, and then cleaned with deionized water to sinter at 500 °C for 30 min. To prepare the mesoporous TiO_2_ layer, a TiO_2_ paste diluted by ethanol of 1:7 weight ratio was spin-coated at 5000 rpm for 40 s and then baked at 500 °C for 30 min. After cooling to room temperature, the TiO_2_ films were immersed into 20 mM TiCl_4_ solution at 70 °C for 30 min and then heated treated at 500 °C for 30 min. A perovskite precursor solution consisting of 461 mg PbI_2_, 160 mg CH_3_NH_3_I dissolving in 1 mL DMF was spin-coated onto the TiO_2_/FTO/glass at 4000 rpm for 20 s. During the spin-coating process, 100 µL toluene was dripped onto the precursor film and then annealed at 100 °C on a hot plate for 10 min. The hole transport solution was prepared by dissolving 72.3 mg spiro-OMeTAD in 1 mL chlorobenzene with additives consisting of 17.5 µL Li-TFSI/acetonitrile (520 mg/mL), 28.8 µL of TBP. The hole transport material was spin-coated at 2000 rpm for 40 s. Finally, an 80 nm Au electrode was deposited on the top of the device using thermal evaporation.

### 2.3. Film and Device Characterization

X-ray diffraction (XRD) test were carried out by an X-ray power diffractometer (SHIMADZU, XRD-6000, Kyoto, Japan Cu Ka radiation, k = 0.15406 nm). The morphological properties of the samples were characterized by the S-4800 scanning electron microscope (SEM). Photocurrent density–voltage (J–V) curves of solar cells were measured with a Keithley 2400 source meter under the simulated AM 1.5G illumination at a calibrated intensity of 100 mW/cm^2^ by a Class AAA solar simulator at room temperature in the air, and the scanning direction is from open circuit at 1.2 V to short circuit at 0 V with a scan rate of 100 mV/s. Steady-state photo-luminescence (PL) spectroscopy was measured with an excitation wavelength of 510 nm. The surface properties of the substrates and perovskite films were analyzed by an Atomic Force Microscopy (Bruker Dimension Icon). The light absorption spectra were recorded on a UV-vis-NIR spectrophotometer (Cary 5000). The steady state and time resolved photoluminescence (TRPL) spectroscopy was measured with a PL spectrometer (F900), and a pulsed laser with a wavelength of 375 nm.

## 3. Results and Discussion

Figure 1a shows the morphology of Ag NPs characterized by SEM. The average diameter of Ag NPs by statistics was 30 nm from in Figure 1b. The extinction spectra of Ag NPs are displayed in Figure 1c, which serves an experimental resonant peak in wavelength 425 nm, that is consistent with the calculated result, analyzed using the finite-difference time-domain (FDTD) method. It is known that the resonance peak of metal nanoparticles such as Au and Ag shift to a longer wavelength with increase in their sizes [27], and the scattering cross-sections of spherical metal nanoparticles increases with size while the light absorption decreases [28]. The Ag NPs embedded into c-TiO_2_ photoanode can enable a longer optical path length by light scattering. Figure 1d displays the electric field distribution of single Ag nanoparticle through theoretical simulation by FDTD method. The plasmon resonance of Ag nanoparticle shows a dipole resonance mode, and the electric field intensity near the nanosphere was enhanced. Thus, incident photos after photoexcitation in Ag NPs were coupled to a conduction band electron, in order to induce collective oscillations of the electrons, which is localized surface plasmon resonances to enhance light absorption in the visible region [29].

**Figure 2:**
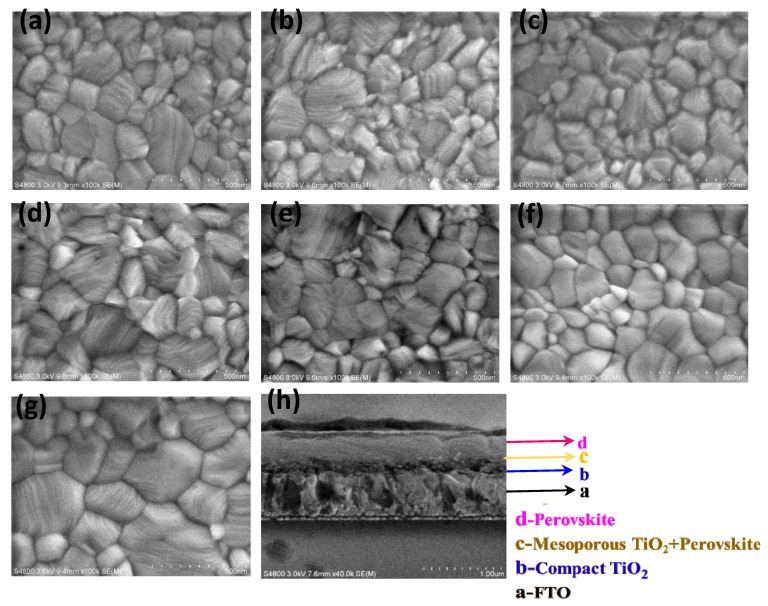
Top-view SEM images of perovskite films on smooth FTO/(**a**–**f**): 0, 5 wt.%, 6.7 wt.%, 10 wt.%, 20 wt.% and 33.3 wt.% of Ag NPs in c-TiO_2_ layer/m-TiO_2_ layer; and (**g**) Top-view SEM image of perovskite film on T-FTO/10 wt.% Ag NPs in c-TiO_2_ layer/m-TiO_2_ layer; (**h**) Cross-sectional SEM image of perovskite film on T-FTO/ 10 wt.% Ag NPs in c-TiO_2_ layer/m-TiO_2_ layer.

In order to study the effect of various mass concentration of Ag NPs in c-TiO_2_ layer in perovskite solar cells (PSCs), we prepared Ag@TiO_2_ with 0 (Pristine), 5 wt.% (S-5 wt.%), 6.7 wt.% (S-6.7 wt.%), 10 wt.% (S-10 wt.%), 20 wt. % (S-20 wt.%) and 33.3 wt.% (S-33.3 wt.%), respectively. Figure 2a–f provides the surface morphology of perovskite films on various Ag NPs in c-TiO_2_ layer. Observed from the SEM image, the pinholes free perovskite film on the m-TiO_2_/c-TiO_2_ with different concentration of Ag NPs/S-FTO were obtained, whose grain size distribution can be seen in Figure S1. The average grain size of CH_3_NH_3_PbI_3_ films on smooth FTO substrates was 200 nm, and the largest grain increased to 300 nm. The perovskite films on T-FTO substrates shown in Figure 2g exhibited similar surface morphology that is continuous and compact, while the larger average grain size of 300 nm and largest grain of 500 nm could be obtained. The thickness of the c-TiO_2_ layer is about 90 nm, as shown in the cross-sectional SEM image in Figure 2h. The CH_3_NH_3_PbI_3_ film of 290 nm thick was fabricated on m-TiO_2_ layer/c-TiO_2_ layer with various wt.% concentrations of Ag NPs/T-FTO. Compared with the ordinary perovskite layer, the thickness of the absorber is reduced [3,30]. The formation of pure CH_3_NH_3_PbI_3_ was obtained by XRD analysis in Figure S2. The scattering peaks attributed to the (110), (220), (310) and (224) planes of CH_3_NH_3_PbI_3_, which indicated that the perovskite film, possess a tetragonal crystal structure.

**Figure 3:**
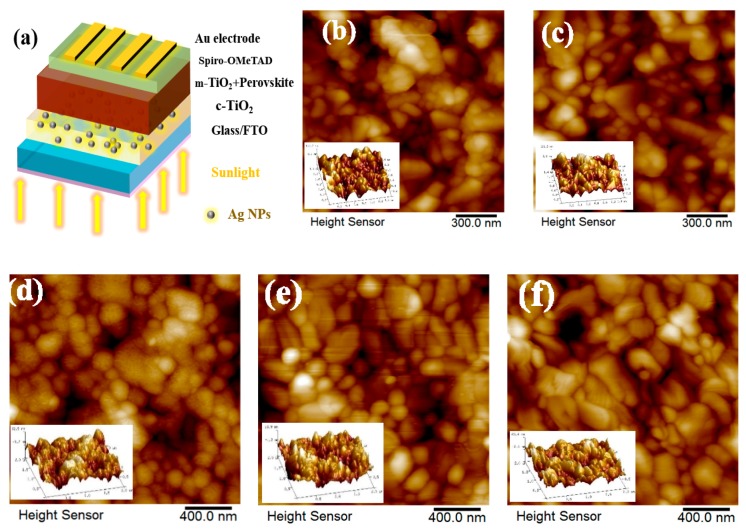
(**a**) Illustration of device structure labeled with different components. Surface morphologies of Atomic Force Microscopy (AFM) images of the S-FTO, T-FTO, perovskite/m-TiO_2_/c-TiO_2_/S-FTO, perovskite/m-TiO_2_/ with Ag NPs concentration of 10 wt.% embedded in c-TiO_2_/S-FTO or T-FTO in (**b**), (**c**), (**d**), (**e**) and (**f**), respectively.

Figure 3a shows the device structure based on light trapping design labeled with different components. Atomic Force Microscopy (AFM) was carried out to analyze the effect of textured morphology on the light trapping and the results shown in Figure 3b,c. The root-mean-roughness (RMS) of smooth Glass/FTO substrates (S-FTO) was 27.9 nm. In comparison, the larger surface roughness of T-FTO was 31.3 nm. The textured morphology of T-FTO substrates contributed to the upper roughness and c-TiO_2_ layer whose RMS was 20.8 nm analyzed from AFM test shown in Figure S3. While the c-TiO_2_ with 10 wt.% Ag NPs/S-FTO and c-TiO_2_/S-FTO have relatively lower RMS values of 19.7 and 19.4 nm, respectively. Figure 3d–f displays the different surface morphology and roughness of the CH_3_NH_3_PbI_3_ layer, where the larger RMS of 12.1 nm for CH_3_NH_3_PbI_3_ film with large-grain perovskite and enhanced film coverage of the underlying rough substrates was achieved through light trapping design. The RMS of the sample with Ag NPs concentration of 10 wt.% on the S-FTO substrates and pristine samples, were 9.3 and 9.7 nm, respectively. Furthermore, an increase in contact area in interface between the textured perovskite film and adjacent charge transport layer is favorable to the higher charge separation efficiencies in a mesoscopic structured perovskite-based solar cell [31].

**Figure 4:**
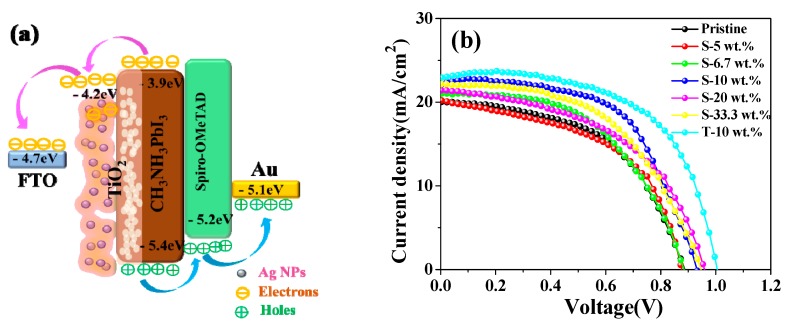
(**a**) The energy level diagram of the PSCs; (**b**) density–voltage (J–V) curves of PSCs at various wt.% concentrations of Ag NPs.

Figure 4a shows an energy level diagram of the perovskite devices based on light trapping design. In a basic PSC, CH_3_NH_3_PbI_3_ photoactive materials absorb photons under illumination to generate electron-hole pair, and then the excitons dissociated at the perovskite/charge transport layer interface. The photogenerated electrons was injected to electron-transporting layer (ETL) to FTO or injection of hole into a hole-transporting materials (HTM) to Au counter electrode. The photocurrent density–voltage (J–V) curves of the plasmonic devices with Ag NPs from 5 to 33.3 wt.% displayed higher short-circuit current density (Jsc), similar or higher power conversion efficiency (PCE), shown in Figure 4b. Specially, the Jsc was increased from 20.03 mA/cm^2^ of the pristine sample to 22.89 mA/cm^2^ with open-circuit voltage (Voc) = 1.02 V and fill factor (FF) = 0.60 of T-10 wt.% sample, and the PCE was 14.01% shown in Table 1, which presents the highest efficiency of perovskite solar cells. The negative work function of TiO_2_ is 4.6 eV, higher than that of Ag NPs (4.26 eV) [32], which can not only act as an electron transporter, but also induce the transition of photoelectrons from Ag NPs to the conduction band of TiO_2_ [24]. It is reported that the Ag NPs in the photovoltaic devices decreased the series resistance and improved the shunt resistance in electrochemical impedance spectroscopy analysis, suggesting an enhanced charge transport with lower charge recombination [33].

**Figure 5:**
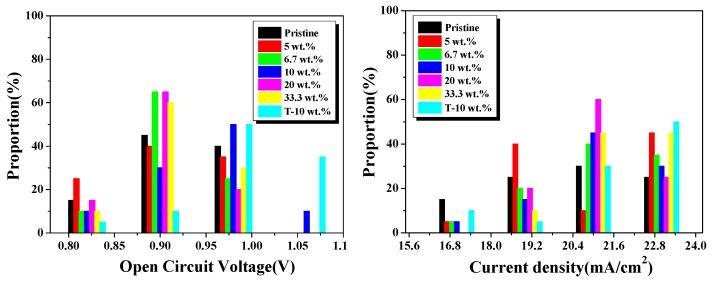

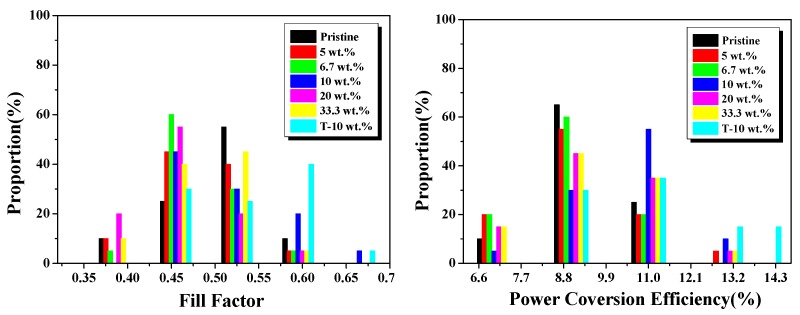
Statistic device performance parameters of Voc, Jsc, FF and power conversion efficiency (PCE) for solar cells with various Ag NPs concentrations.

On the other hand, Ag NPs with low dielectric losses and strong extinction coefficients for other metal nanoparticles can increase the far-field scattering in LSPR effect, which extends the optical path length and increases the total light trapping in devices. Mie theory describes the extinction behavior of spherical metal nanoparticles when excited with an incident electric field [34], in which light scattering properties induced by metal nanostructures with sizes of more than 30 nm can achieve ideal solar harvesting. When plasmonic nanostructures are excited by the incident light, the electromagnetic field with higher energy is formed, that contributes to more electron-hole generation. The reproducibility of the perovskite solar cells by light trapping design is shown in Figure 5 and Table S1, where the S-10 wt.% devices exhibit an average PCE of 10.42% with average value of Voc = 0.95 V, Jsc = 21.37 mA/cm^2^ and FF = 0.50; whereas, in the case of the T-10 wt.%, devices show a PCE of 11.73% with Voc = 0.99 V, Jsc = 21.61 mA/cm^2^, and the PCE errors of perovskite solar cells mainly comes from the Jsc values of devices. The increase is due to the LSPR effect of Ag NPs incorporated into TiO_2_ and enhanced charge collection on the textured substrates. However, the PSCs with Ag NPs concentration of 20 wt.% and 33.3 wt.% showed lower photoelectric conversion efficiency. This is because the larger number of Ag NPs act as charge-trap sites to provide recombination centers. Thus, these mechanisms occur simultaneously in the PSCs. Meanwhile, the T-10 wt.% device has a lower hysteresis effect than that of the pristine sample (Figure S4), which may be due to the light trapping design. For a long stability test (Figure S5), the perovskite devices are stored in air at room temperature without encapsulation. The PCE of the pristine device obviously decreased and these devices with Ag NPs were more stable, which might be ascribed to the light trapping effect. However, the specific mechanism is not clear and needs further study.

**Figure 6:**
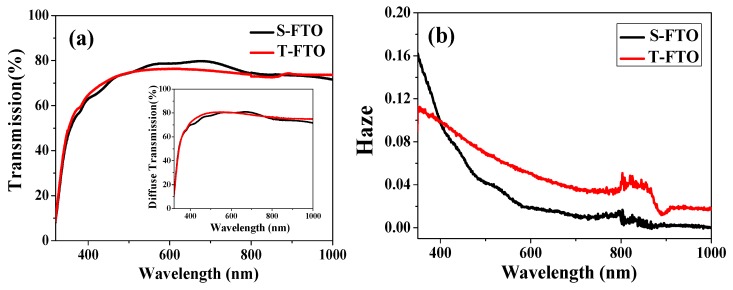

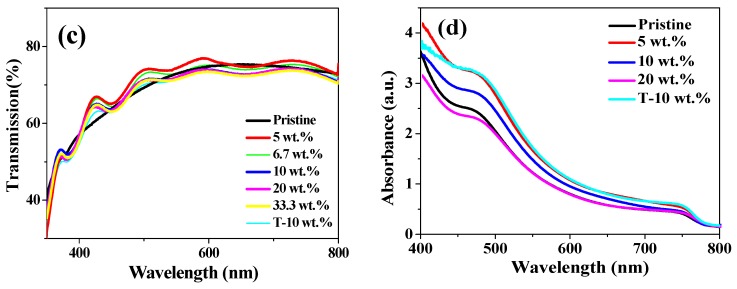
(**a**) The transmission spectra of different substrates; (**b**) Haze curves of S-FTO and T-FTO substrates; (**c**) The transmission spectra of various Ag NPs wt.% from 0 to 33.3% in TiO_2_ films on S-FTO and T-FTO; (**d**) UV-vis absorbance data of FTO/ with or without Ag NPs concentration embedded in c- TiO_2_/m-TiO_2_/ CH_3_NH_3_PbI_3._

To further illustrate the light trapping effect designed from Ag NPs and texture FTO substrates, we performed Haze property of the S-FTO and T-FTO in Figure 6b, which was obtained by a corresponding calculation, based on transmission and diffuse transmission measurement shown in Figure 6a. There is little difference in transmittance between the two substrates, but the haze of textured FTO is greater than that of smooth FTO in the visible range. Figure 6c shows the transmission spectra of various Ag NPs wt.% from 0 to 33.3% in TiO_2_ films on S-FTO and T-FTO. A downward peak around 440 nm was due to the localized plasmonic excitation of Ag NPs embedded into the TiO_2_ films. It can be seen that the total light transmittance of TiO_2_ layer with and without Ag NPs/FTO substrate had slight change. However, the UV-vis absorption spectra of FTO/ with or without Ag NPs concentration embedded in c-TiO_2_/m-TiO_2_/ CH_3_NH_3_PbI_3_ shows obvious variation features. Accordingly, the difference of light absorption in the spectra shown in Figure 6d is mainly due to the light trapping effect from the Ag particles and textured substrates. The absorption in the region of wavelengths from 410 to 500 nm is enhanced for Ag NPs samples, corresponding to the plasmonic resonance region of Ag NPs shown in Figure 1a. The T-10 wt.% sample shows a higher absorption at the wavelength range of 500–800 nm than that of samples embedded in different Ag NPs concentration, and also higher than the devices without Ag NPs, which is in good agreement with the Haze curves. The presence of highly polarizable Ag nanoparticles improved the radiative decay of excitons and increased optical path lengths through light scattering [35]. The S-5 wt.% sample had lower absorption in the wavelength from 400 to 500 nm, which may be the cause of the decreased efficiency in S-5 wt.% PSCs. Although the S-20 wt.% sample displayed a higher light absorption than the S-10 wt.% sample, the S-20 wt.% PSCs, in the end, had lower efficiencies. It is due to more charge recombination sites caused by the large amount of Ag NPs; therefore, the textured substrates and appropriate Ag nanoparticles helped to enhance the light trapping effect.

**Figure 7:**
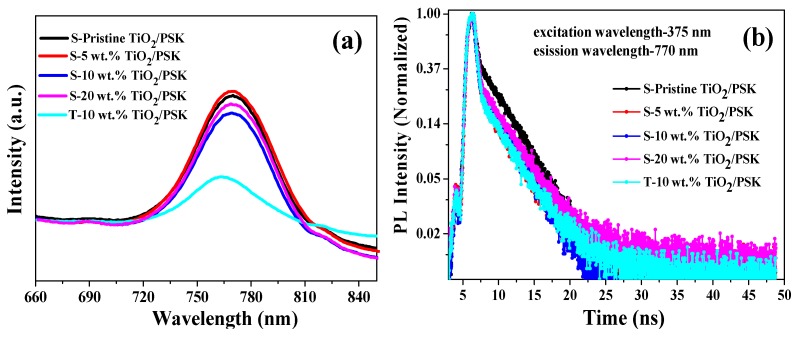
Carrier transfer characterization of (**a**) Steady state photo-luminescence (PL) spectra and (**b**) time-resolved PL spectra for perovskite (PSK)/m- TiO_2_ without or with Ag NPs concentration of 5 wt.%, 10 wt.% and 20 wt.% embedded in c- TiO_2_/ FTO.

Figure 7a presents the steady state photoluminescence (PL) spectroscopy based on perovskite/ TiO_2_. The neat perovskite film sample was prepared by one anti-solvent method in Device fabrication section. The measurements show that the intensity of the emission peak at 770 nm was reduced after incorporating Ag NPs owing to the c-TiO_2_/m-TiO_2_ quenches the perovskite steady-state PL. The intensity of the emission peak 770 nm was drastically reduced for the T-10 wt.% sample, suggesting that the better excitation separation at the TiO_2_/perovskite interface. Therefore, Ag NPs are helpful for the improvements of charge generation and collection. To further explain the increased photocurrent density and rapid charge collection, the time resolved photoluminescence (TRPL) was useful for quantitative information on the yield of light-induced charge separation in Figure 7b. A bi-exponential function was used to fit the PL decay time as follows [36]:

(1) $$I(t)=A1\exp(\frac{t}{\tau 1})+A2\exp(\frac{t}{\tau 2})$$

where *τ*_1_ is the time constants for the fast process, which shows the fast decay of the quenching of free carriers from the perovskite to the electron transport layer, while *τ*_2_ is related to the slow decay of the radiative decay. The average lifetime can be calculated by the following equation [37]:

(2) $$\bar{\tau }=\frac{A_{1}\tau_{1}^{2}+A_{2}\tau_{2}^{2}}{A_{1}\tau_{1}+A_{2}\tau_{2}}$$

The results are shown in Table 2. The average lifetime of perovskite solar cells with Ag NPs concentration from 5 wt.% to 20 wt.% were lower than the pristine sample, the $\bar{\tau }$ of which was 3.356 ns. *A*_1_ and *A*_2_ are the coefficient of the corresponding component with the defined fluorescence lifetime. Meanwhile, the lifetimes can be correlated to the rate for the interfacial electron transfer process based on the following equation:

*k*_et_ = 1/*τ*_ads_ − 1/*τ*_un_ (3)

where *τ*_ads_ and *τ*_un_ denote the emission lifetimes for the CH_3_NH_3_PbI_3_/TiO_2_ and for the neat CH_3_NH_3_PbI_3_, and the *k*_et_, are the special rate constant and average lifetime for the charge injection process [30]. The *k*_et_ values of Ag NPs in TiO_2_ samples are higher than that of the TiO_2_ sample without Ag NPs. The electron injection rate of CH_3_NH_3_PbI_3_/S-5 wt.%TiO_2_, CH_3_NH_3_PbI_3_/S-10 wt.%TiO_2_, CH_3_NH_3_PbI_3_/S-20 wt.%TiO_2_, CH_3_NH_3_PbI_3_/T-10 wt.%TiO_2_ were 2.049 × 10^9^ s^−1^, 2.131 × 10^9^ s^−1^, 1.814 × 10^9^ s^−1^, 1.931 × 10^9^ s^−1^, respectively, which are faster than the CH_3_NH_3_PbI_3_/ TiO_2_ without Ag NPs interface of 1.825 × 10^9^ s^−1^. A lower *τ*_2_ values for the Ag NPs in TiO_2_ samples with the Ag NPs concentrations of 5 wt.%, and 10 wt.% were also found, whereas the S-20 wt.% sample had the highest *τ*_1_ value of 0.491 ns, and other parameter values are listed in Table 2. From this study we can reasonably infer that the appropriate Ag NPs amount is an effective way to enhance charge generation and collection so that the trap sites might be reduced in TiO_2_, and then increased photocurrent density can be observed in PSCs.

## 4. Conclusions

Through the light trapping design, PSCs based on textured the textured Glass/FTO and Ag NPs embedded into TiO_2_ layer have been successfully fabricated. The perovskite film based on textured substrates shows lager grain size and textured micromorphology. The device with textured substrates and incorporated with Ag NPs has a higher power conversion efficiency of 14.01%, showing an improvement of 33.7% over that of a device without Ag NPs based on smooth substrates. The improvement is not only attributed to the enhancement of light absorption, but also contributes to better excitation separation. Therefore, light trapping design based on the optical properties illuminates possibilities for improving the performance of perovskite solar cells.

## Figures and Tables

**Table 1:** Photovoltaic performances summary of the perovskite devices.

Samples	Voc (V)	Jsc(mA/cm^2^)	FF	PCE (%)
Pristine	0.91	20.03	0.51	9.29
S-5 wt.%	0.90	20.21	0.50	9.13
S-6.7 wt.%	0.92	21.03	0.51	9.72
S-10 wt.%	0.94	22.08	0.59	12.23
S-20 wt.%	0.97	21.41	0.49	10.18
S-33.3 wt.%	0.95	22.15	0.52	10.93
T-10 wt.%	1.02	22.89	0.60	14.01

**Table 2:** The calculating parameters of the perovskite samples with various Ag NPs concentration in TiO_2_ from the time resolved photoluminescence (TRPL) measurement.

Samples	*τ*_1_ (ns)	*A*_1_	*τ*_2_ (ns)	*A*_2_	*k*_et_ (10^9^ s^−1^)	$\bar{\tau }$ (ns)
Pristine	0.486	0.0396	4.306	0.0135	1.825	3.356
S-5 wt.%	0.440	0.0638	4.472	0.0067	2.049	2.522
S-10 wt.%	0.423	0.0587	4.290	0.0084	2.131	2.712
S-20 wt.%	0.491	0.0524	4.493	0.0087	1.814	2.904
T-10 wt.%	0.464	0.0627	4.459	0.0067	1.931	2.488

## Supplementary Materials

The following are available online at http://www.mdpi.com/2079-4991/8/10/815/s1, Figure S1: Grain size distribution of perovskite films on m-TiO_2_ layer/(a)–(f): 0, 5 wt.%, 6.7 wt.%, 10 wt.%, 20 wt. % and 33.3 wt.% of Ag NPs in c-TiO_2_ layer/S-FTO, and (g) Grain size distribution of perovskite films on m-TiO_2_ layer/c-TiO_2_ layer with 10 wt.% Ag NPs/T-FTO. Figure S2: XRD patters of the perovskite layers on FTO/TiO_2_ with various Ag NPs concentrations of 0, 5 wt.%, 6.7 wt.%, 10 wt.%, 20 wt.%, 33.3 wt.%. Figure S3: Surface morphologies of AFM images of (a) c-TiO_2_ /S-FTO and (b) c-TiO_2_ after embedding 10 wt.% Ag NPs/S-FTO (c) c-TiO_2_ after embedding 10 wt.% Ag NPs /T-FTO. Figure S4: The device performance under forward scan and reverse scan of (a) pristine sample and (b) T- 10 wt.% sample. Figure S5: The stability test of PSCs without or with Ag NPs concentration from 5 wt.% to 20 wt.% without encapsulation conditions. Table S1: The average photovoltaic performances summary of the perovskite devices.
