# Supplementary material for: Light Trapping Effect in Perovskite Solar Cells by the Addition of Ag Nanoparticles, Using Textured Substrates
Source: Nanomaterials (Basel). 2018 Oct 10;8(10):815. doi: 10.3390/nano8100815 (PMC6215094; doi:10.3390/nano8100815)
Supplement: Supplementary file 1 [file nanomaterials-08-00815-s001.pdf]

nanomaterials

Light Trapping Effect in Perovskite Solar Cells by the Addition of Ag Nanoparticles, using Textured Substrates

Jiabin Hao [1,2], Huiying Hao [1,*], Jianfeng Li [1], Lei Shi [1], Tingting Zhong [1], Chen Zhang [1], Jingjing Dong [1], Jie Xing [1], Hao Liu [1] and Zili Zhang [1]

[1] School of Science, China University of Geosciences, Beijing 100083, China
[2] School of Materials Science and Technology, China University of Geosciences, Beijing 100083, China
* Correspondence: huiyinghaol@cugb.edu.cn

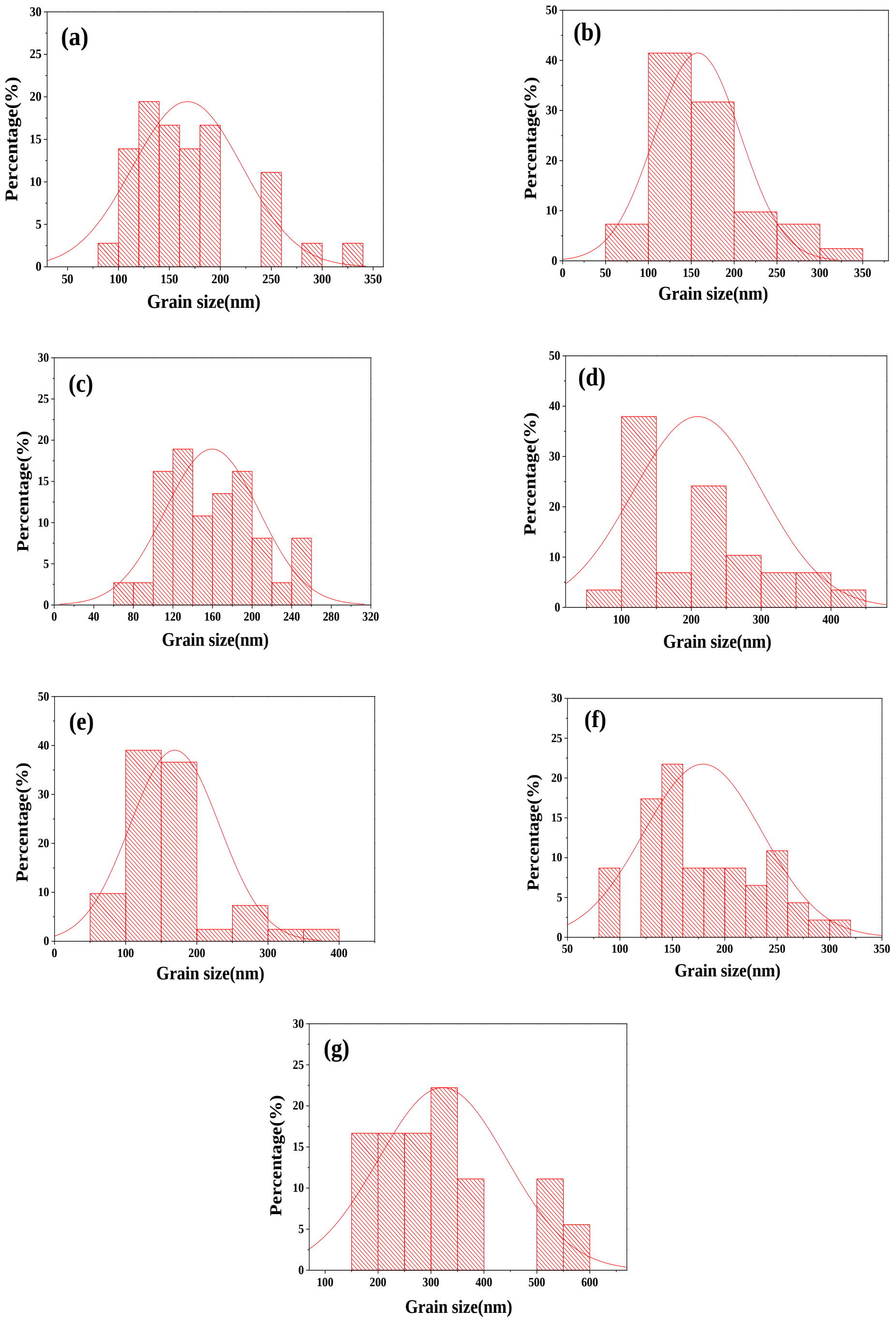

Figure S1. Grain size distribution of perovskite films on TiO_2 layer embedded Ag NPs of (a)–(g): 0, 5 wt.%, 6.7 wt.%, 10 wt.%, 20 wt. %, 33.3 wt.% and 10 wt.%, (a)–(f) upper films are on S-FTO and (g) on T-FTO.

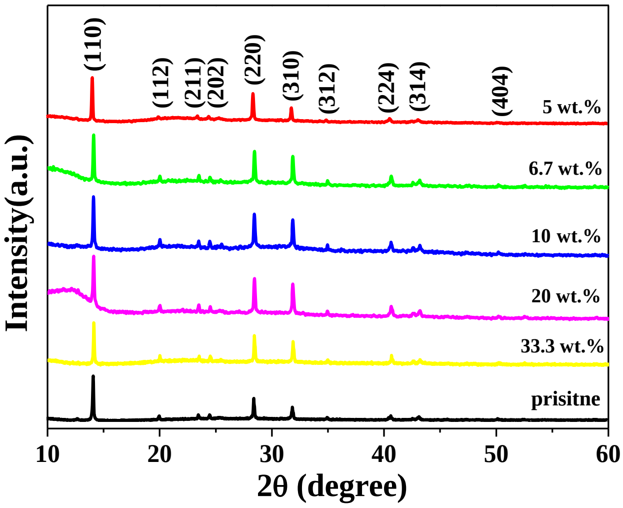

Figure S2. XRD patters of the perovskite layers on FTO/TiO_2 with various Ag NPs concentrations of 0, 5 wt.%, 6.7 wt.%, 10 wt.%, 20 wt.%, 33.3 wt.%.

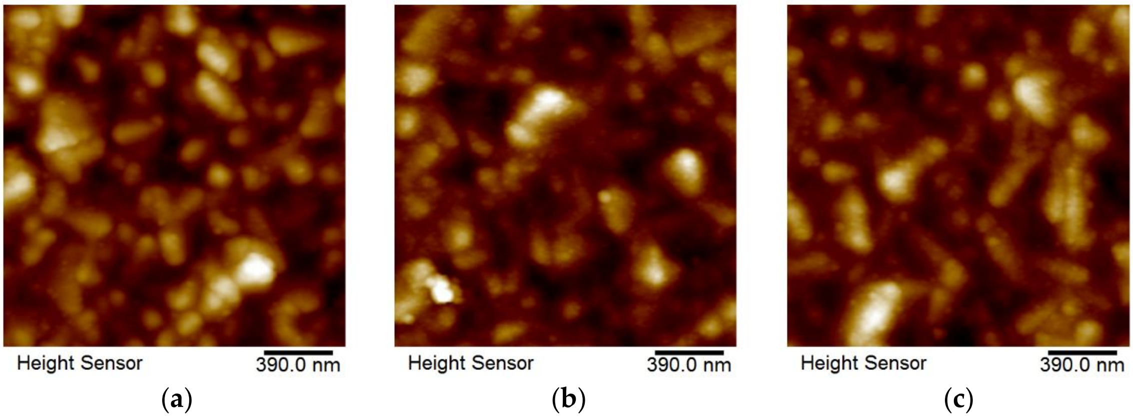

Figure S3. Surface morphologies of AFM images of (**a**)c-TiO_2 /S-FTO and (**b**) c-TiO_2 after embedding 10 wt.% Ag NPs /S-FTO (**c**) c-TiO_2 after embedding 10 wt. % Ag NPs /T-FTO.

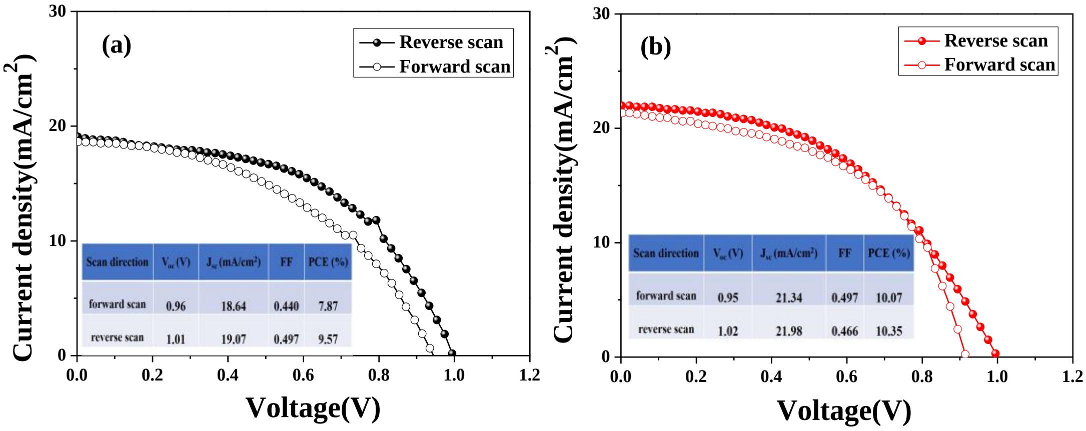

Scan direction	V_{oc} (V)	J_{sc} (mA/cm^2)	FF	PCE (%)
forward scan	0.96	18.64	0.440	7.87
reverse scan	1.01	19.07	0.497	9.57

Scan direction	V_{oc} (V)	J_{sc} (mA/cm^2)	FF	PCE (%)
forward scan	0.95	21.34	0.497	10.07
reverse scan	1.02	21.98	0.466	10.35

Figure S4. The device performance under forward scan and reverse scan of (**a**)pristine sample and (**b**) T- 10 wt.% sample.

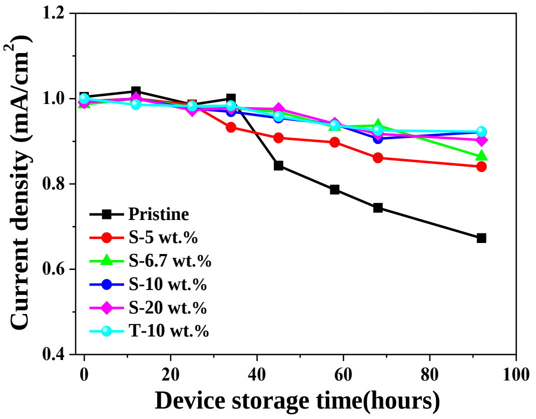

Figure S5. The stability test of PSCs without or with Ag NPs concentration from 5 wt.% to 20 wt.% without encapsulation conditions.

Table S1. The average photovoltaic performances summary of the perovskite devices.

Samples	V_{oc} (V)	J_{sc} (mA/cm^2)	FF	PCE (%)
Pristine	0.93 ± 0.06	20.12 ± 2.24	0.49 ± 0.06	9.18% ± 0.99
S-5 wt.%	0.92 ± 0.06	20.99 ± 2.11	0.48 ± 0.05	9.19% ± 1.30
S-6.7 wt.%	0.91 ± 0.04	21.06 ± 1.74	0.47 ± 0.04	9.03% ± 1.16
S-10 wt.%	0.95 ± 0.06	21.37 ± 1.80	0.50 ± 0.06	10.42% ± 1.41
S-20 wt.%	0.92 ± 0.05	21.39 ± 1.37	0.47 ± 0.05	9.37% ± 1.25
S-33.3 wt.%	0.92 ± 0.05	21.42 ± 1.26	0.48 ± 0.05	9.43% ± 1.32
T-10 wt.%	0.99 ± 0.06	21.61 ± 1.93	0.54 ± 0.07	11.73% ± 2.28
